# Continuous Quinacrine Treatment Results in the Formation of Drug-Resistant Prions

**DOI:** 10.1371/journal.ppat.1000673

**Published:** 2009-11-26

**Authors:** Sina Ghaemmaghami, Misol Ahn, Pierre Lessard, Kurt Giles, Giuseppe Legname, Stephen J. DeArmond, Stanley B. Prusiner

**Affiliations:** 1 Institute for Neurodegenerative Diseases, University of California, San Francisco, California, United States of America; 2 Department of Neurology, University of California, San Francisco, California, United States of America; 3 Department of Pathology, University of California, San Francisco, California, United States of America; University of Edinburgh, United Kingdom

## Abstract

Quinacrine is a potent antiprion compound in cell culture models of prion disease but has failed to show efficacy in animal bioassays and human clinical trials. Previous studies demonstrated that quinacrine inefficiently penetrates the blood-brain barrier (BBB), which could contribute to its lack of efficacy *in vivo*. As quinacrine is known to be a substrate for P-glycoprotein multi-drug resistance (MDR) transporters, we circumvented its poor BBB permeability by utilizing MDR^0/0^ mice that are deficient in *mdr1a* and *mdr1b* genes. Mice treated with 40 mg/kg/day of quinacrine accumulated up to 100 µM of quinacrine in their brains without acute toxicity. PrP^Sc^ levels in the brains of prion-inoculated MDR^0/0^ mice diminished upon the initiation of quinacrine treatment. However, this reduction was transient and PrP^Sc^ levels recovered despite the continuous administration of quinacrine. Treatment with quinacrine did not prolong the survival times of prion-inoculated, wild-type or MDR^0/0^ mice compared to untreated mice. A similar phenomenon was observed in cultured differentiated prion-infected neuroblastoma cells: PrP^Sc^ levels initially decreased after quinacrine treatment then rapidly recovered after 3 d of continuous treatment. Biochemical characterization of PrP^Sc^ that persisted in the brains of quinacrine-treated mice had a lower conformational stability and different immunoaffinities compared to that found in the brains of untreated controls. These physical properties were not maintained upon passage in MDR^0/0^ mice. From these data, we propose that quinacrine eliminates a specific subset of PrP^Sc^ conformers, resulting in the survival of drug-resistant prion conformations. Transient accumulation of this drug-resistant prion population provides a possible explanation for the lack of *in vivo* efficacy of quinacrine and other antiprion drugs.

## Author Summary

Prion diseases belong to the class of neurodegenerative disorders that include Alzheimer, Parkinson and Huntington diseases. In each of these disorders, a specific protein in the brain changes shape and accumulates, leading to neuronal loss and damage. These diseases are uniformly fatal after a period of neurodegeneration, dementia and motor dysfunction. Quinacrine, an antimalarial drug, is able to eliminate prions from dividing cells in culture, yet is ineffective in diseased mice and human patients. Here, we provide an explanation for this failure. Our data indicate that the administration of quinacrine results in the proliferation of drug-resistant prions. This insight will enable us to develop more effective antiprion therapeutics in the future.

## Introduction

Prion diseases are a class of rare, neurodegenerative disorders that include Creutzfeldt-Jakob disease (CJD) and kuru in humans, BSE in cattle, and scrapie in sheep [1]. They are uniformly fatal and follow a rapid clinical course after an extended asymptomatic incubation period. Prion diseases are caused by an unconventional transmissible pathogen called a prion that is devoid of nucleic acids [2]. Prions are composed entirely of an abnormally folded conformer of the prion protein and replicate by catalyzing the conversion of the endogenous, cellular prion protein (PrP^C^) into an aberrantly folded conformation (PrP^Sc^) [1],[3],[4],[5]. Although PrP^Sc^ accumulation has been demonstrated to be the primary pathogenic event in prion disease, the exact molecular and cellular mechanisms of its formation and the ensuing neurodegeneration remain largely unknown.

To date, no therapeutic agent against prion diseases has been identified. Numerous compounds have been found to have antiprion activity in cell culture models of prion disease, including pentosan polysulfate, dextran sulfate, HPA-23, Congo red, suramin, dendritic polyamines and quinacrine, among others; for a comprehensive review, see [6]. However, none of these compounds have been shown to be broadly effective against a range of prion strains in animal models when administered at a post-symptomatic clinical stage, and none have been shown to prolong the disease course in human clinical trials.

Among the antiprion compounds, quinacrine seemed to be the most promising for immediate application in the treatment of prion disease because it has been used for decades as an antimalarial drug [7]. In two independent studies, incubation of persistently prion-infected neuroblastoma cells with quinacrine induced the clearance of protease-resistant PrP^Sc^ [8],[9]. A subsequent study using bis-acridine compounds, which are comprised of two acridine ring scaffolds connected by a linker, demonstrated improved potency [10]. Despite its efficacy in cell-culture models, quinacrine administration *in vivo* has not appeared to be promising. Collins and colleagues reported that the incubation time of mice intracerebrally inoculated with prions and subsequently treated with quinacrine via gavage feeding did not differ from that of untreated mice [11]. In a second study, intraperitoneally administered quinacrine failed to extend the survival time of prion-infected mice [12]. In human clinical studies, quinacrine has had mixed success. In some patients, treatment with quinacrine transiently altered the clinical course of disease, whereas in others it failed to improve the clinical outcome [13] (Geschwind et al., unpublished results).

In wild-type mice, quinacrine inefficiently penetrates the blood-brain barrier (BBB). After an oral dose of 40 mg/kg/day, quinacrine accumulates in the brain at concentrations of less than 1 µM ([14]; Ahn et al., manuscript in preparation; this study). The quinacrine concentrations needed for half-maximal depletion of PrP^Sc^ (EC_50_) in cell culture were reported to be ∼1 µM and ∼7 µM for extracellular and intracellular accumulation, respectively [9],[15]. Thus, the failure of quinacrine *in vivo* seemed likely to result from its insufficient accumulation in the brain.

The finding that the P-glycoprotein transporter is involved in the efflux of quinacrine across the BBB [14],[16] opened another avenue of investigation. Pgp is encoded by two genes in humans (*MDR1*[ABCB1] and *MDR2*[ABCB4]) and three genes in rodents (*mdr1a*, *mdr1b* and *mdr2*) [17]. Pgp is a member of a large family of ATP-binding cassette (ABC) transporters, 12 of which have been implicated in some form of drug resistance [17],[18]. Pgp encoded by *MDR1* in humans, and *mdr1a* and *mdr1b* in mice, confers the vast majority of observed multidrug resistance. Hereafter in this paper, “Pgp” will refer to the proteins encoded by this class of MDR genes (there is no current evidence that *mdr2* is involved in quinacrine efflux). Given that quinacrine is a substrate for Pgp, we tested whether deletion of the *mdr* genes (knockout MDR1a/1b (−/−, −/−), denoted as MDR^0/0^) would improve the pharmacological profile of quinacrine and enhance its *in vivo* efficacy.

Here, we report that quinacrine can accumulate in the brains of orally treated MDR^0/0^ mice at concentrations exceeding 100 µM. Despite attaining these high concentrations in the brain, quinacrine failed to extend the survival times of prion-inoculated MDR^0/0^ mice. The results suggest that the failure of quinacrine *in vivo* cannot be attributed solely to its pharmacokinetic properties. Quinacrine treatment transiently reduced PrP^Sc^ levels in the brains of mice, but PrP^Sc^ levels recovered during the course of treatment. Parallel results in cell culture suggest that continuous quinacrine treatment leads to the selective survival of drug-resistant prion conformations. However, these resistant conformations do not stably propagate in the absence of quinacrine.

## Results

### Effect of quinacrine treatment on the survival of prion-inoculated FVB, CD-1 and MDR^0/0^ mice

We evaluated whether quinacrine treatment would extend the survival time of prion-infected mice of three genetic backgrounds: CD1 (wild-type outbred), FVB (wild-type inbred) and MDR^0/0^. Mice were inoculated intracerebrally with 30 µL of 1% brain homogenate containing the Rocky Mountain Laboratory (RML), rodent-passaged, scrapie prion strain. An oral regimen of 40 mg/kg/day of quinacrine was initiated at selected time-points [0, 30, 60, 70, 80, 95, and 105 d postinoculation (dpi)] and continued for different time intervals (10, 20, 30, 50, 60 d, or remaining lifetime of the mouse). The mice were monitored for neurological dysfunction and sacrificed upon the onset of prion disease. At least 9 mice were allowed to reach the endpoint of disease for each arm of the experiment and used to determine the mean incubation period **(** **Table 1** **)**. For some arms, several mice were sacrificed during the course of the experiment in order to measure quinacrine uptake, PrP^Sc^ levels, and neuropathologic changes (see below). Treated mice will be referred to as S[a-b] or S[a>], for which “S” is the mouse strain, “a” indicates the initiation of quinacrine in dpi, “b” indicates the cessation of treatment in dpi, and “>” indicates lifelong treatment.

**Table 1:** Incubation periods for prion-infected MDR^0/0^, FVB and CD1 mice orally treated with 40 mg/kg/day of quinacrine.

Mouse line	Treatment initiated (dpi)	Treatment duration (d)	Incubation period (days±SEM)
MDR^0/0^	30	60	135±4
	30	30	143±1 *
	60	30	128±3
	60	20	141±2 *
	60	10	141±2 *
	60	until death	115±2
	0	until death	115±4
	untreated	-	124±3
FVB	60	30	128±1 *
	60	until death	118±2
	untreated	-	121±2
CD1	105	until death	121±2
	95	30	129±5
	95	until death	129±3
	80	30	116±4
	80	until death	121±2
	70	30	145±5 *
	70	60	131±5
	70	until death	121±2
	untreated	-	127±2

*: *Statistically significant (P<0.01) prolongation of the incubation period compared to untreated controls. For all experiments, n = 9 mice.*

We did not observe obvious signs of acute toxicity resulting from quinacrine treatment in any of the experimental arms. Quinacrine treatment did not substantially alter the progression of disease **(** **Table 1** **)**. Short-term quinacrine treatment (≤30 d) resulted in statistically significant increases in the incubation periods (P<0.01) for five experimental arms **(** **Table 1** **)**. However, the magnitudes of these effects were small and all mice developed neurologic dysfunction. Together, our data indicate that quinacrine is largely inefficacious in all prion-inoculated mice observed.

### Quinacrine accumulation in treated, RML-inoculated FVB and MDR^0/0^ mice

**Figure 1:**
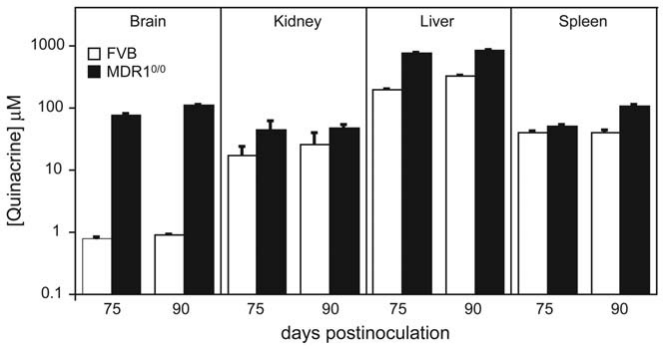
Accumulation of quinacrine in treated FVB and MDR^0/0^ mice. The levels of quinacrine in the brains, kidneys, livers and spleens of FVB[60–90] (white bars) and MDR^0/0^[60–90] (black bars) were measured at 75 and 90 dpi by quantitative LC/MS/MS and comparison to known standards. The error bars indicate the standard error of the mean (*n* = 3).

We assessed the accumulation of quinacrine in the brains, kidneys, livers and spleens of FVB[60–90] and MDR^0/0^[60–90] mice at 75 and 90 dpi by quantitative LC/MS/MS and comparison to known standards **(** **Fig. 1** **)**. Three mice were analyzed for each measurement. At 75 dpi, the concentration of quinacrine in the brains of FVB and MDR^0/0^ mice were ∼1 µM and ∼100 µM, respectively. Quinacrine concentrations were also greater in all other tissues of MDR^0/0^ mice compared to FVB mice. Similar trends were observed in tissues obtained at 90 dpi. In treated MDR^0/0^ mice, quinacrine was distributed throughout the brain and was readily detected in the hippocampus, cortex, thalamus, cerebellum, and brainstem (Ahn et al., manuscript in preparation).

### Neuropathology of quinacrine-treated, RML-inoculated MDR^0/0^ mice

**Figure 2:**
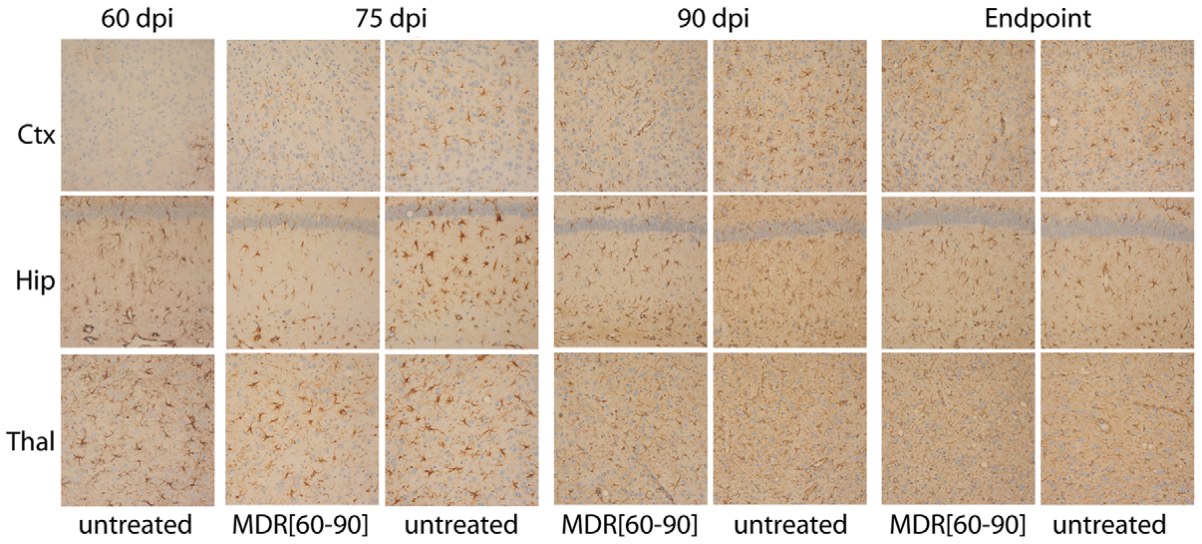
Immunohistochemical analyses of brain sections from quinacrine-treated, RML-infected MDR^0/0^[60–90] mice. Brain sections were prepared from treated and untreated MDR^0/0^ mice that were sacrificed 60, 75, and 90 dpi as well as from mice at the endpoint of disease. Sections were taken from nearly identical regions of the cortex (ctx), hippocampus (hip) and thalamus (thal), then immunostained for the glial fibrillary acidic protein (GFAP) to visualize astrocytic gliosis. Scale bar represents 50 µm and applies to all panels.

In parallel, we examined neuropathologic changes in prion-infected MDR^0/0^[60–90] mice and untreated controls at 75 and 90 dpi **(** **Fig. 2** **)**. The brains of infected MDR^0/0^ mice showed mild, focal astrocytic gliosis in the thalamus and hippocampus before quinacrine was administered, which was also observed with 15 d of quinacrine treatment (75 dpi). In comparison, the brains of untreated mice at 75 dpi showed moderate to severe astrocytic gliosis in the thalamus and hippocampus, and mild astrocytic gliosis in the cortex. At 90 dpi, astrocytic gliosis in the brains of treated and untreated mice showed subtle differences. At the endpoint of disease, astrocytic gliosis and spongiform degeneration were similar for both treated and untreated MDR^0/0^ mice. Together, these data suggest that quinacrine has a slight effect on neuropathologic changes upon initial administration, but does not halt widespread neurodegeneration during the course of disease.

### PrP^Sc^ accumulation in the brains of quinacrine-treated, RML-inoculated MDR^0/0^ mice

**Figure 3:**
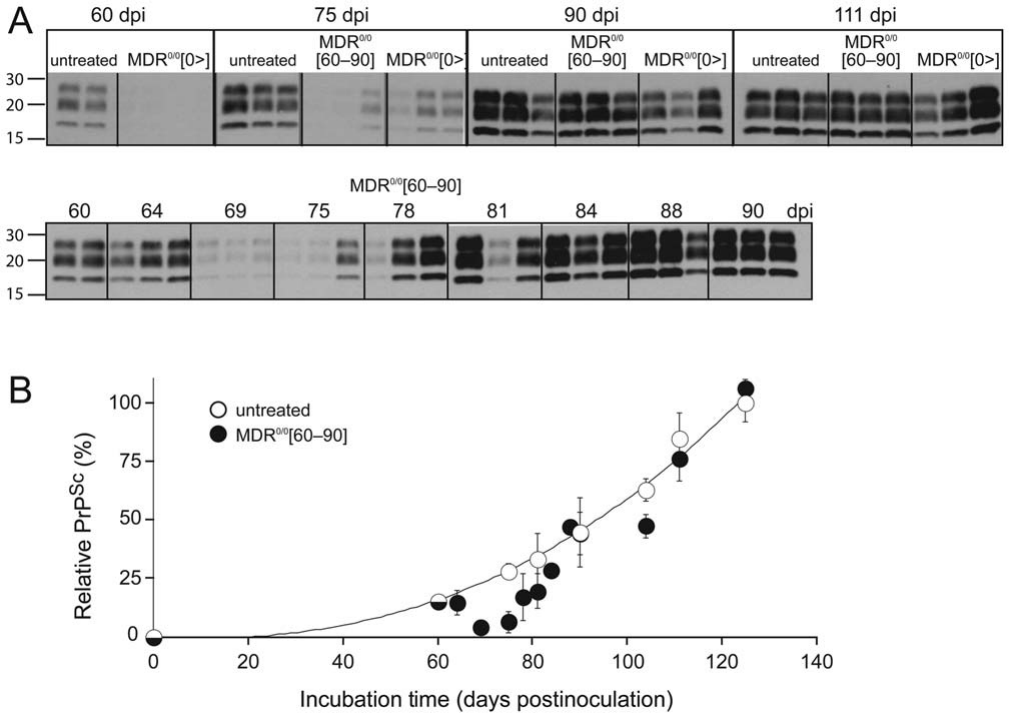
PrP^Sc^ levels in the brains of quinacrine-treated, RML-infected MDR^0/0^ mice. **(A)** Western blots show PrP^Sc^ levels in the brains of untreated, MDR^0/0^[0>], and MDR^0/0^[60–90] mice that were sacrificed at 60, 75, 90, and 111 dpi. PrP^Sc^ accumulation was also probed in infected MDR^0/0^[60–90] mice sacrificed at 64, 69, 78, 81, 84, and 88 dpi. Extracts were normalized with respect to the total protein concentration and digested with PK, as described in *Materials and Methods*. Lanes represent brain homogenates of individual mice. Apparent molecular masses based on the migration of protein standards are shown in kilodaltons. **(B)** The kinetics of PrP^Sc^ accumulation in the brains of untreated MDR^0/0^ and treated MDR^0/0^[60–90] mice analyzed quantitatively by ELISA. Error bars represent the standard error of the mean (*n* = 3).

We analyzed the kinetics of PrP^Sc^ accumulation during the incubation period of MDR^0/0^[60–90], MDR^0/0^[0>], and untreated mice. Two or three animals were sacrificed at 60, 75, 90 and 111 dpi, and the accumulation of protease-resistant PrP^Sc^ in the brain was analyzed by Western immunoblotting **(** **Fig. 3A** **)** and quantitative ELISA **(** **Fig. 3B** **)** following proteinase K (PK) digestion. Additionally, PrP^Sc^ levels were measured in MDR[60–90] mice at 7 other time-points (64, 69, 75, 78, 81, 84, and 88 dpi). At 60 dpi, MDR^0/0^[0>] mice had lower PrP^Sc^ levels compared to untreated controls. At 75 dpi, PrP^Sc^ levels were lower in both MDR^0/0^[0>] and MDR^0/0^[60–90] mice compared to untreated controls. However, this difference was not evident at later time-points even with continuous quinacrine administration (MDR[0>] mice). In the absence of quinacrine treatment, PrP^Sc^ was readily detectable at 60 dpi. With quinacrine treatment, PrP^Sc^ signals were faint but detectable beginning at 75 dpi, a delay of 15 d compared with no treatment. In MDR^0/0^[60–90] mice, PrP^Sc^ was abundant before treatment was initiated (60 dpi), but barely detectable at 69 dpi, indicating that 9 d of quinacrine treatment reduced PrP^Sc^ in the brain. These data indicate that quinacrine can both delay the formation and induce the clearance of PrP^Sc^. However, in both cases, PrP^Sc^ eventually accumulated in the brains of quinacrine-treated animals until they showed neurologic signs of illness. The transient reduction of PrP^Sc^ was not observed in FVB[60–90] mice (data not shown), indicating that accumulation of quinacrine to high levels in the brain is required for prion reduction.

### Conformational stability and CDI analysis of PrP^Sc^ in the brains of quinacrine-treated, RML-inoculated MDR^0/0^ mice

While quinacrine did not halt the accumulation of PrP^Sc^ in the brains of infected mice, we asked if whether quinacrine treatment altered the biochemical characteristics of PrP^Sc^. We prepared 10% brain homogenates of inoculated, untreated MDR and chronically treated MDR[0>] mice at the endpoint of disease. We exposed the samples to increasing concentrations of guanidinium hydrochloride (GdnHCl) for 1 h followed by PK digestion, then quantified the levels of protease-resistant PrP^Sc^ by ELISA **(** **Table 2** **)**. As an additional control, we also analyzed untreated brains spiked with 100 µM of quinacrine. PrP^Sc^ in the brains of quinacrine-treated mice was slightly less stable than in the brains of untreated controls. With 1.3 M GdnHCl, a significantly greater fraction of quinacrine-exposed PrP^Sc^ was denatured compared to quinacrine-naive PrP^Sc^ (*n* = 3; P<0.01). We also analyzed PrP^Sc^ in these brain homogenates using another method, the conformation-dependent immunoassay (CDI) [19]. This assay measures the differential ability of PrP^Sc^ conformations to bind anti-PrP antibodies by measuring the bound ratio between native and denatured samples following partial purification by phosphotungstate (PTA). The CDI measurement for PrP^Sc^ purified from quinacrine-treated brains was substantially different from untreatedbrains spiked with quinacrine (*n* = 3; P<0.01, **Table 2**). Together, these findings suggest that quinacrine exposure altered the physical properties of PrP^Sc^ accumulating in MDR[0>] mice.

**Table 2:** Relative conformational stability and immunospecificity of PrP^Sc^ in the brains of MDR^0/0^ mice continuously treated with quinacrine compared to untreated controls.

	Relative PrP^Sc^ (%)		
GdnHCl (M)	Untreated MDR^0/0^	Untreated MDR^0/0^ + 100 µM QA	MDR^0/0^[0>]
0	100.0±0.0	100.0±5.0	100.0±0.0
0.5	92.2±5.6	85.8±6.1	88.1±6.1
1.0	53.8±8.5	48.6±3.0	44.3±2.6
1.3	21.7±7.1	26.0±0.7 *	5.9±1.3 *
1.5	4.5±1.9	10.2±0.6	2.3±3.4
1.7	1.5±1.7	7.2±0.4	0.5±0.9
2.0	0.0±0.0	0.2±0.1	0.2±0.3
**CDI (F_D_/F_N_)**	19.6±0.1	18.3±0.1 *	14.0±0.2 *

*At the endpoint of disease, brain homogenates were exposed to the indicated concentrations of GdnHCl for 1 h, then to 50 µg/mL of PK for 1 h at 37°C. As an additional control, 100 µM quinacrine was added to untreated brain homogenates to mimic the quinacrine content of the treated brain homogenate. The levels of protease-resistant PrP^Sc^ were quantitatively measured by ELISA and normalized to 0 M GdnHCl. CDI measurements are the ratio of ELISA signals from PTA-precipitated native and denatured PrP^Sc^ as described in* Materials and Methods. Values are mean percentages ± standard error (n = 3). * P<0.01.

### Second passages of quinacrine-treated PrP^Sc^

We next determined whether the physically altered, quinacrine-treated PrP^Sc^ could cause disease upon transmission to MDR^0/0^ mice. Brain homogenates from infected, untreated MDR^0/0^ and chronically treated MDR[0>] mice at the endpoint of disease were inoculated into MDR^0/0^ mice. The mice were monitored for neurological dysfunction and sacrificed upon the onset of prion disease. The mean incubation periods for MDR^0/0^ mice infected with untreated and quinacrine-treated inocula were 112±3 and 116±1 days, respectively (*n* = 8). Analyses of PrP^Sc^ in 10% brain homogenates of these ill mice, using GdnHCl denaturation and CDI as described above, showed no significant differences in the stabilities and CDI measurements **(** **Table 3** **)**, suggesting that the physically altered, quinacrine-induced PrP^Sc^ is not a stably propagating strain.

**Table 3:** Relative conformational stability and immunospecificity of PrP^Sc^ in the brains of MDR^0/0^ mice after inoculation with untreated and MDR^0/0^[0>] homogenates.

	Relative PrP^Sc^ (%)	
GdnHCl (M)	Untreated MDR^0/0^ inoculum	MDR^0/0^[0>] inoculum
0	100.0±5.0	100.0±6.0
0.5	100.0±8.0	102.0±7.0
1.0	63.8±4.4	55.3±4.8
1.3	31.3±1.1	29.1±1.9
1.5	12.1±1.4	15.7±1.9
1.7	5.6±0.0	5.0±1.8
2.0	0.0±0.0	0.2±0.1
**CDI (F_D_/F_N_)**	19.5±0.1	18.5±0.1

*At the endpoint of disease, brain homogenates were exposed to the indicated concentrations of GdnHCl for 1 h, then to 50 µg/mL of PK for 1 h at 37°C. The levels of protease-resistant PrP^Sc^ were quantitatively measured by ELISA and normalized to 0 M GdnHCl. CDI measurements are the ratio of ELISA signals from PTA-precipitated native and denatured PrP^Sc^ as described in* Materials and Methods. *Values are mean percentages ± standard error (n = 3).*

### Creation of quinacrine-resistant PrP^Sc^ in cultured cells

**Figure 4:**
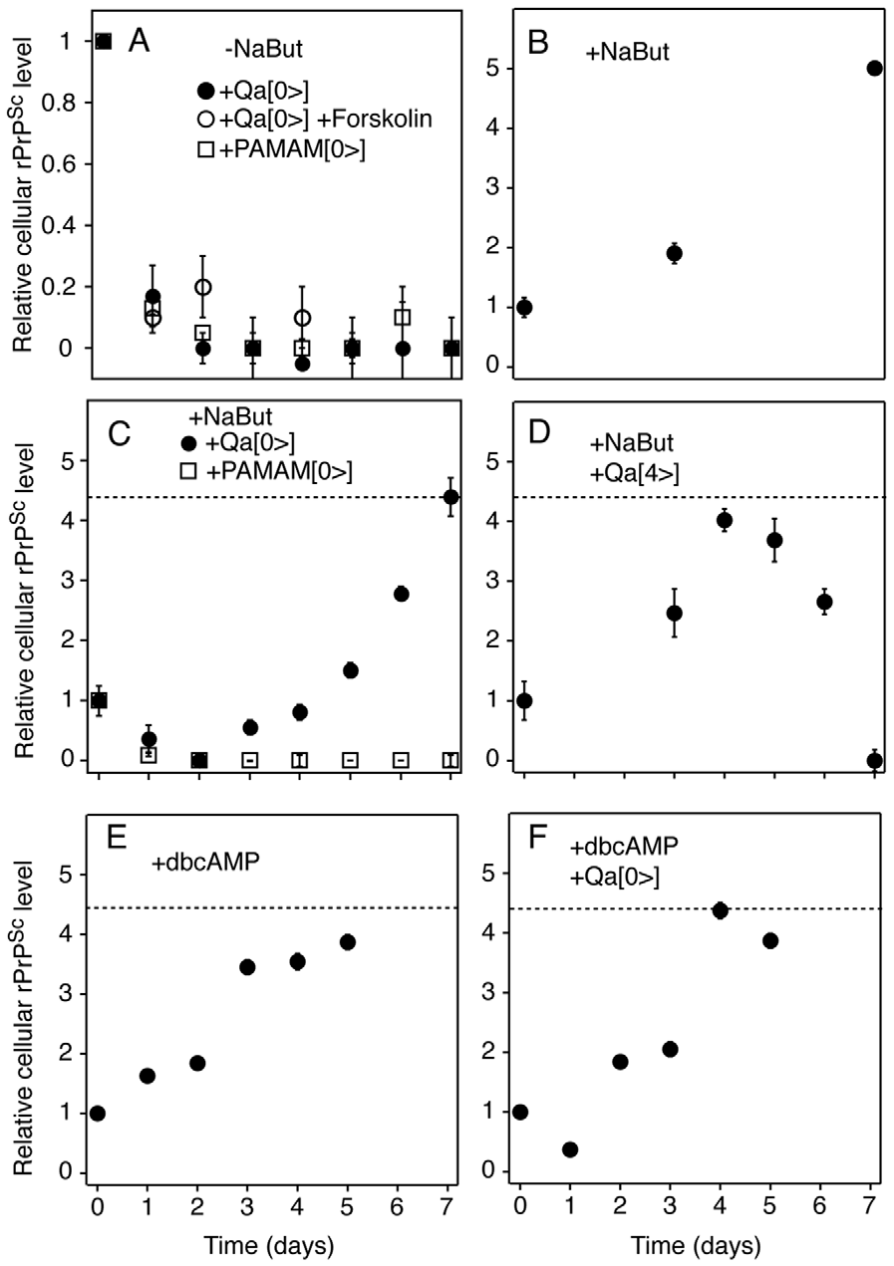
Effect of quinacrine on PrP^Sc^ levels in division-arrested ScN2a-cl3 cells. Prion-infected ScN2a-cl3 cells were either cultured normally (–NaBut, panel A), or in the presence of 10 mM sodium butyrate (+NaBut, panels B–D) or 5 mM dibutyryl cAMP (+dbcAMP, panels E–F) in order to induce differentiation. **(A)** Dividing cells were treated with 10 µg/mL PAMAM (open squares) or 1 µM quinacrine beginning at day 0 (+Qa[0>]) in the presence (open circles) or absence (filled circles) of 1 µM forskolin. Sodium butyrate–treated cells were **(B)** left untreated, **(C)** incubated with 10 µg/mL PAMAM or 1 µM quinacrine beginning at day 0, or **(D)** treated with 1 µM quinacrine after splitting on day 4 (+Qa[4>]). Dibutyryl cAMP–treated cells were **(E)** left untreated or **(F)** incubated with 1 µM quinacrine beginning at day 0. Cells were fed with media and compounds (as required by the experiment) every 2 d. Every day, cells were harvested, lysed, digested with PK and the relative PrP^Sc^ levels were measured by ELISA. The dashed lines represent the relative level of PrP^Sc^ in a fully confluent ScN2a-cl3 culture. The error bars represent the standard error of the mean of three independent experiments.

We next asked if quinacrine-resistant PrP^Sc^ could be formed in cell culture. The re-appearance of drug-resistant PrP^Sc^ upon continuous addition of quinacrine to prion-infected N2a cell lines (ScN2a) has not been previously observed. Past experiments were conducted in continuously dividing cultures. Here, we examined the kinetics of PrP^Sc^ clearance in infected cells after differentiation by exposure to sodium butyrate or dibutyryl cAMP [20],[21] **(** **Fig. 4** **)** to exclude the effect of cell division on prion levels [22]. PrP-overexpressing ScN2a-cl3 cells were plated at 70% confluency in the presence of 10 mM sodium butyrate or 5 mM dibutyryl cAMP. The cultures were either left untreated (-Qa), or exposed to 1 µM quinacrine beginning at day 0 (Qa[0>]) or day 4 (Qa[4>]). In a control experiment, cells were cultured in the absence of differentiating agents and treated with 1 µM quinacrine beginning at day 0. After each day, cells were lysed and the relative amount of PK-resistant PrP^Sc^ was measured by ELISA.

In dividing ScN2a-cl3 cultures, quinacrine rapidly cleared PrP^Sc^ and suppressed its accumulation for at least 7 d **(** **Fig. 4A** **)**. In sodium butyrate–treated cultures, PrP^Sc^ levels were transiently reduced by exposure to quinacrine, but increased after 2 d of treatment **(** **Fig. 4C** **)**. The observed PrP^Sc^ re-accumulation in ScN2a-cl3 cells is similar to that observed in treated MDR^0/0^ mice and suggests that PrP^Sc^ becomes resistant to the effects of quinacrine upon continuous treatment. Using similar treatment, drug resistance does not develop for an alternative antiprion compound, the polyamidoamine (PAMAM) G4 [23] **(** **Fig. 4A, C** **)**. Quinacrine proved similarly ineffective when infected cells were division-arrested by dibutyryl cAMP **(** **Fig. 4F** **)**. This effect of dibutyryl cAMP is not dependent on activation of the PKA pathway, as the addition of forskolin does not result in a rebound in PrP^Sc^ levels **(** **Fig. 4A** **)**. In differentiated cells exposed to sodium butyrate for 4 d prior to quinacrine administration, PrP^Sc^ levels only decreased after exposure to quinacrine, indicating that observed changes resulted from quinacrine and not from sodium butyrate exposure **(** **Fig. 4D** **)**. PrP^Sc^ slowly increased in untreated, differentiated cells to levels similar to an untreated confluent culture **(** **Fig. 4B, 4E** **)**. Unlike the *in vivo* experiments, the PrP^Sc^ that accumulated in division-arrested cells after the addition of quinacrine did not have a significantly altered conformational stability (data not shown). Thus, PrP^Sc^ can become quinacrine-resistant without a change in conformational stability as detected by melting of the protein structure with GdnHCl.

Upon addition of sodium butyrate and dibutyryl cAMP, N2a cells became differentiated and could not be further passaged. Instead, in an attempt to propagate quinacrine-treated PrP^Sc^, we tried to infect N2a cells with lysates from treated cells (data not shown) but were unable to achieve infection. However, lysates from control untreated, infected cells were able to infect N2a cells. We also were unsuccessful at infecting N2a cells with homogenate from the brains of quinacrine-treated mice (data not shown). The difference in infectivity between treated and untreated samples might indicate a conformational change in PrP^Sc^ induced by quinacrine. However, given that infection of cell lines with prions is a stochastic phenomenon, we cannot rule out the possibility that the difference in infectivity was caused by chance.

## Discussion

Quinacrine is a potent antiprion drug in culture but has failed to slow the course of disease in CJD patients and experimental mouse models of prion disease. It is unclear whether this translational gap is due primarily to quinacrine's pharmacokinetic or pharmacodynamic properties *in vivo*. As a minimum requirement for *in vivo* efficacy, quinacrine must accumulate in the brain at concentrations that exceed its *in vitro* effective concentration. In chronically treated wild-type mice, the highest nontoxic tolerable dose of quinacrine is 40 mg/kg/day (Ahn et al., manuscript in preparation). At this dose, quinacrine accumulates in the brains of FVB mice at a concentration of ∼1 µM. In continuously dividing, ScN2a cells, the EC_50_ of quinacrine is also ∼1 µM [9]. However, this represents the nominal effective concentration of quinacrine added to the culture media. When N2a cells are exposed to quinacrine, the intracellular concentration of quinacrine is typically 30 to 50 times higher than its extracellular concentration [15] (Ghaemmaghami et al., unpublished results). Thus, the actual EC_50_ of quinacrine at its site of action may be much greater than its reported EC_50_ value of 1 µM. We therefore reasoned that the efficacy of quinacrine *in vivo* might be improved by increasing its steady-state accumulation in the brain to levels approaching its intracellular effective concentration. Because the efflux of quinacrine from the brain is governed by Pgp, an mdr-associated protein [14],[16], active transport can be inhibited by the deletion of the *mdr1a* and *mdr1b* genes (MDR^0/0^) or the co-administration of Pgp inhibitors [18]. Here, we orally administered quinacrine to MDR^0/0^ mice and achieved drug levels in the brain that were nearly two orders of magnitude higher than those in FVB mice. This level of accumulation far exceeds the intracellular EC_50_ of quinacrine in culture. Despite its excess accumulation, quinacrine again failed to extend the survival time of prion-inoculated mice. The results indicate that quinacrine's lack of efficacy *in vivo* is not solely due to its pharmacokinetic properties and may represent a pharmacodynamic failure. The data are consistent with a previous study reporting that continuous intraventricular administration into the cerebral ventricle does not improve the efficacy of quinacrine [24].

A recent study reported a decrease in the expression of cerebovascular Pgp in a CJD patient, suggesting a possible role for Pgp in prion pathogenesis [25]. However, we did not observe a significant difference between the incubation periods of inoculated, untreated MDR^0/0^ and wild-type mice. These results suggest that Pgp alone does not significantly influence the accumulation of PrP^Sc^.

Kinetic analysis of PrP^Sc^ levels following drug administration provided insight into the basis of quinacrine's failure *in vivo*. In the brains of prion-inoculated MDR^0/0^ mice, PrP^Sc^ levels diminished upon the initiation of quinacrine treatment, remained low for several days, but gradually increased despite the continuous presence of the drug. There are two potential explanations for this trend: (1) target tissues, such as the brain, alter their responsiveness to quinacrine or (2) prions become resistant to the actions of the drug. There is precedence for the former phenomenon. It has been shown that during chronic administration, various drugs and steroid hormones lose efficacy by inducing the expression of efflux transformers and cytochrome P450 [26],[27],[28]. However, we did not observe a measurable decrease in brain quinacrine levels during the course of treatment (**Fig. 1**; Ahn et al., manuscript in preparation). Therefore, we propose that chronic quinacrine treatment resulted in the formation of drug-resistant PrP^Sc^ conformations that survived the initial treatment. A similar linkage between limited efficacy and the formation of drug-resistant prions was cited for amyloidophilic compounds [29]. The following two observations are consistent with this hypothesis. First, we observed that PrP^Sc^ accumulating in the brains of chronically treated mice had a lower conformational stability compared to that of untreated controls, suggesting that quinacrine induced a structural change within the prion population. Second, we were able to induce the formation of quinacrine-resistant prions in cell culture, indicating that drug resistance can be induced outside the context of the central nervous system.

Interestingly, the formation of quinacrine-resistant prions is only observed in division-arrested cultured cells. The steady-state concentration of PrP^Sc^ in a cell is established by the balance between its rates of formation and clearance. As has been noted before [22],[30], the apparent clearance rate for PrP^Sc^ in a dividing cell is the sum of the rate of catabolism and the rate of cell division. Thus, the apparent rate of clearance for a given prion conformation is likely to be slower in stationary cells compared to dividing cells. In a dividing cell, the process of cell division artificially enhances the rate of clearance and prevents the accumulation of PrP^Sc^. Therefore, the probability that a partially resistant conformation survives quinacrine treatment is increased in stationary cells. These observations suggest that conducting drug screens in stationary cells may be more likely to identify antiprion compounds that prove effective *in vivo*.

Increasing evidence shows that prion-infected tissues harbor multiple, distinct PrP^Sc^ conformations [31],[32],[33],[34],[35],[36]. Thus, in an infected brain, each prion strain may have a different degree of susceptibility to the actions of quinacrine. In this context, the administration of quinacrine could provide selective pressure for the selection of drug-resistant conformations. Indeed, the PrP^Sc^ rebound observed in this study is reminiscent of the failure of antiviral drugs caused by selection of drug-resistant variants. However, unlike resistant viral strains, the quinacrine-resistant conformation formed here was not able to propagate in the absence of the drug and thus cannot be considered a stably propagating strain. In the absence of quinacrine, the initial physical properties of the prion population are rapidly re-established. In viral therapy, the administration of drug cocktails, combining compounds with differing modes of action, reduces the probability of virologic failure. Ultimately, the co-administration of multiple antiprion compounds may be required in order to avoid the formation of resistant prion conformations. Additionally, targeting PrP^C^, the endogenous substrate shared by all prion strains, is likely to identify compounds that have broad specificity and are uniformly effective against all strains.

## Materials and Methods

### Materials

Quinacrine dihydrochloride, PAMAM G4, forskolin and sodium butyrate were purchased from Sigma-Aldrich (St. Louis, MO). N2a cells were obtained from American Tissue Culture Collection. Minimal essential medium (MEM) with Earle's salts; Dulbecco's Modified Eagle Medium (DMEM) High Glucose 1× with 4.5 g/L D-glucose and L-glutamine and without sodium pyruvate; cell dissociation buffer; fetal bovine serum (FBS); Geneticin (50 mg/mL); penicillin-streptomycin (10,000 units/mL and 10,000 µg/mL, respectively); and GlutaMAX were purchased from Gibco. Dithiothreitol (DTT; 0.5 M 10×); 4× loading buffer; and proteinase K (PK) were purchased from Invitrogen. Complete protease inhibitor (PI) cocktail tablets were from Roche Diagnostics. Western blotting detection reagents 1 and 2 were from GE Healthcare. Anti-PrP antibodies Fab D18 [37] and Fab D13 conjugated to horseradish peroxidase (HRP) [38] were prepared as previously described.

### Animals and tissue preparation

All protocols were approved by the University of California San Francisco Animal Care and Use Committee. Approximately five-week-old male and female wild-type (FVB and CD1) and MDR^0/0^ mice were purchased from Charles River (Wilmington, MA) and Taconic (Germantown, NY), respectively. Mice were inoculated intracerebrally with 30 µL of 1% brain homogenate containing the RML prion strain. Treatment consisted of 40 mg/kg/day of quinacrine in a chocolate-flavored liquid diet [39] administered beginning at 0, 30, and 60 dpi for MDR^0/0^ mice; 60 dpi for FVB mice; and 70, 80, 95, and 105 dpi for CD1 mice. The initiation of quinacrine treatment varied for each mouse strain to reflect the relative incubation period of the respective mouse line.

At 75 and 90 dpi, 2–3 mice were euthanized. The left half-brain was snap frozen on powdered dry ice for quinacrine extraction and biochemical analysis; the right half-brain was fixed for pathological analysis. Brain homogenates (10% w/v) were prepared using a Precellys 24 homogenizer (Bertin Technologies, Montigny-le-Bretonneux, France) in pure distilled water (Invitrogen, San Diego, CA) and were aliquoted and stored at −80°C.

Animals were observed every day for signs of neurologic disease. Mice were diagnosed with prion disease when they exhibited three or more of the following neurological symptoms: ataxia, circling, depression, and blank stare. Upon diagnosis, mice were killed and their brains collected as described above for analysis.

### Measurement of quinacrine concentration in tissues

Quinacrine was extracted from brain samples and its concentration was measured by LC/MS/MS as described previously [14] (Ahn et al., manuscript in preparation). Briefly, working solution containing 10 µg/mL of quinacrine was diluted with 10% tissue homogenates from untreated, control animals to make 500 µl of 1 µg/mL of standard 1 solution for each tissue type. The standard 1 solution was then serially diluted two-fold. Nine standard solutions (200 µl each) and a blank solution (0 µg/mL) were frozen at −80°C. A total of 400 µl of acetonitrile containing the internal standard (50 ng/mL of chlorophenamine) was added to 200 µl of 10% tissue homogenates or standard solutions. The samples were vortexed vigorously twice for 1 min and centrifuged at 16,000 *g* for 5 min. LC/MS/MS system consisted of Shimadzu LC-10 AD pumps, a Waters Intelligent Sample Processor 717 Plus autosampler, and a Micromass Quattro LC Ultima triple quadruple tandem mass spectrometer. The mass spectrometer was set to electrospray ionization in the positive-ion mode. Quinacrine and its metabolites, O-demethylated quinacrine (M1) and mono-desethyl quinacrine (M2), were monitored by multiple-reaction monitoring (MRM) at 400.5>142.2 m/z for QA, 384.5>142.2 m/z for M1, 372.2>114.2 m/z for M2, and 277.2>142.2 m/z for internal standard (chlorphenamine). The column was a Betasil C18 column (50×4.6 mm) from Hypersil-Keystone and the mobile phase consisted of CH_3_OH/H_2_O/trifluoroacetic acid (45∶55∶0.05) with 1 mM ammonium formate. The flow rate was 0.8 mL/min.

### Measurement of protease-resistant PrP^Sc^

Ten percent brain homogenates (100 µL) were diluted with 1 mL lysis buffer and digested with 20 µl of 1 mg/mL PK at 37°C for 1 h. PMSF (final concentration of 1 mM) was added to stop the digestion. Sample volumes of 200 µl were used for ELISA analysis and of 800 µl for Western blot analysis.

ELISA plates were prepared as described previously [22]. Triplicate samples (200 µL each) were transferred to a 96-well PCR plate and 50 µl of 6 M GdnHCl (Pierce, Rockford, IL) was added to each sample. Samples were heated to 85°C for 15 min and 5 µl of samples were added onto an ELISA plate containing 245 µl of 1% BSA per well. Samples were incubated at 4°C overnight. The next day, ELISA plates were washed 3 times with TBST buffer (10 mM Tris/HCl, pH 8.0; 150 mM NaCl; 0.5% Tween-20). After washes, 100 µl of D13-HRP antibody in 1% BSA (1∶1000 dilution) was added to each well and plates were incubated at 37°C for 1 h. Subsequently, plates were washed 7 times with TBST and 100 µl of 2,2′-azino-bis(3-ethylbenzthiazoline-6-sulfonic acid (ABTS) was added to each well. After developing for 15 to 20 min, plates were read using a SpectraMax Plus microplate reader running SoftMaxPro (Molecular Devices, Sunnyvale, CA). In each plate, standard ladders of recombinant mouse PrP and uninoculated brain samples were placed as controls.

For Western blot analysis, PrP^Sc^ pellets from 800 µl of PK-digested samples were collected and resuspended with lysis buffer. SDS sample running buffer and reducing reagent (Invitrogen) were added. Samples were heated to 95°C for 5 min and run in a 4–12% Tris-glycine SDS gel (Invitrogen). The gel was transferred to PVDF membrane using an iBlot (Invitrogen) and the membrane was blocked with 5% milk for 30 min. The membranes were subsequently incubated overnight with D13-HRP antibody and washed 3 times with TBST for 5 min before developing with ECL reagent.

### Conformational-stability assay

Brain homogenates (10 µL) were incubated with GdnHCl, in a range of 0 to 2 M, at 22°C for 2 h. The samples were subsequently diluted with lysis buffer to a final concentration of 0.4 M of GdnHCl. PK was added at a final concentration of 50 µg/mL and the samples were incubated at 37°C for 1 h. PMSF (final concentration of 1 mM) was added to stop PK digestion. The samples were subsequently analyzed by ELISA as described above.

### Conformation-dependent immunoassay

Each sample was divided into two aliquots, precipitated by the addition of 1% PTA and resuspended in lysis buffer. The first aliquot was untreated and designated native; the second aliquot was mixed to a final concentration of 4 M GdnHCI, heated for 20 min at 80°C, and designated denatured. Both samples were diluted 20-fold with lysis buffer containing protease inhibitors (5 mM PMSF; aprotinin and leupeptin, 4 g/ml each). Then, 200 µl volumes were loaded on 96-well polystyrene plates that were previously coated with the mAb D18. The plates were blocked overnight at 18–22°C in 0.2 M phosphate buffer, pH 7.2, and then blocked with TBST, pH 7.2, containing 0.25% BSA (w/v) and 6% Sorbitol (w/v), and 0.03% (w/v) NaN_3_. The plates were washed 3× with TBS, pH 7.8, containing 0.05% (v/v) of Tween 20, then incubated at room temperature for 2 h. HRP-labeled D13 anti-PrP antibody was added and incubated as described [33]. The plates were developed by the addition of ABTS (2,2′-azino-di 3-ethylbenzthiazoline-6-sulfonate) after seven washing steps with TBST (Tris-buffered saline Tween-20) and the signals were measured. After normalization, the ratios of antibody binding to denatured versus native aliquots were calculated.

### Pathology

After mice were euthanized, their right half-brains were fixed in 10% formalin for a minimum of 3 d. Fixed brains were processed and embedded in paraffin, and 8-µm sections were cut from four representative brain regions: cortex, cerebellum, hippocampus and thalamus. Slides were deparaffinized and endogenous peroxidases blocked with 3% H_2_O_2_ in methanol for 20 min. The slides were washed 3 times for 5 min using PBS with 0.1% Tween 20 (PBST) with 3 buffer changes. The glial fibrillary acidic protein (GFAP) target antigen does not require retrieval for antibody recognition (rabbit polyclonal anti-GFAP, Dako). Nonspecific antibody binding was blocked with 5% normal goat serum (NGS) in PBST for 30 min, then the slides were incubated with the primary antibody at 1∶1000 in PBST at 4°C overnight. After washing, the sections were incubated with a biotinylated goat anti-rabbit secondary antibody (Pierce) at 1∶1000 in PBST with 5% NGS for 1 h at room temperature. After additional washing, the Vector ABC kit was used per the manufacturer's instructions. Sections were washed and developed with the Vector DAB kit for 3 min and washed again. Tween 20 was not present in the third wash. The sections were counterstained for 10 s in hematoxylin (Fisher), taken through graded alcohols to xylene, and coverslipped using Permount (Fisher). Images were taken at 20× and 40× magnifications using the SpotFlex camera and program on the Leica DM-IRB microscope.

### Time-course experiments of quinacrine treatment in cultured cells

The PrP-overexpressing N2a-cl3 cell line was created by stably transfecting N2a cells with the pSPOX.neo vector expressing full-length mouse PrP under the control of the hCMV promoter with DOTAP liposomal transfection reagent (Roche). Stably transfected lines were cloned by serial dilution and 15 individual clones were assayed for PrP expression level by Western immunoblotting. The N2a-cl3 clone overexpresses PrP at ∼6× the levels of untransfected N2a cells. N2a-cl3 cells were infected with RML prions as previously described [40] and subcloned to produce ScN2a-cl3 cells. ScN2a-cl3 cells were maintained at 37°C in MEM supplemented with 10% FBS and 1% GlutaMAX (Invitrogen) in 100-mm plates and fed with fresh media every 2 d. Approximately 70% confluent cells were plated and 1 µM quinacrine or 10 µg/mL PAMAM G4 was added in the presence of 10 mM sodium butyrate or 5 mM dibutyryl cAMP for stationary conditions. Dividing cells were plated at 10% confluency in the presence of 1 µM quinacrine, 10 µg/mL PAMAM G4 and/or 1 µM forskolin (and absence of sodium butyrate or dibutyryl cAMP) and split 1∶10 when they become confluent. At the end of each day for 7 d, cells were washed with PBS and harvested using cell dissociation buffer (Invitrogen). Cells were lysed with lysis buffer (100 mM Tris/HCl, pH 8.0; 150 mM NaCl; 0.5% NP-40; 0.5% sodium deoxycholate) and protein concentrations were measured using a bicinchoninic acid protein assay kit (Fisher, Rockford, IL). Protein extracts were normalized to 1 mg/mL total protein with lysis buffer prior to PK digestion and ELISA analysis.
